# Graph analysis of β_2_ adrenergic receptor structures: a “social network” of GPCR residues

**DOI:** 10.1186/2193-9616-1-16

**Published:** 2013-12-05

**Authors:** Samuel Sheftel, Kathryn E Muratore, Michael Black, Stefano Costanzi

**Affiliations:** Department of Chemistry, American University, 4400 Massachusetts Ave, Northwest, Washington, DC 20016 USA; Department of Computer Science, American University, Northwest, Washington, DC 20016 USA; Center for Behavioral Neuroscience, American University, Northwest, Washington, DC 20016 USA

**Keywords:** G protein-coupled receptor (GPCR), β_2_ adrenergic receptor (β_2_ AR), Graph theory, Networks, Closeness-centrality, Non-covalent interactions

## Abstract

**Purpose:**

G protein-coupled receptors (GPCRs) are a superfamily of membrane proteins of vast pharmaceutical interest. Here, we describe a graph theory-based analysis of the structure of the β_2_ adrenergic receptor (β_2_ AR), a prototypical GPCR. In particular, we illustrate the network of direct and indirect interactions that link each amino acid residue to any other residue of the receptor.

**Methods:**

Networks of interconnected amino acid residues in proteins are analogous to social networks of interconnected people. Hence, they can be studied through the same analysis tools typically employed to analyze social networks – or networks in general – to reveal patterns of connectivity, influential members, and dynamicity. We focused on the analysis of closeness-centrality, which is a measure of the overall connectivity distance of the member of a network to all other members.

**Results:**

The residues endowed with the highest closeness-centrality are located in the middle of the seven transmembrane domains (TMs). In particular, they are mostly located in the middle of TM2, TM3, TM6 or TM7, while fewer of them are located in the middle of TM1, TM4 or TM5. At the cytosolic end of TM6, the centrality detected for the active structure is markedly lower than that detected for the corresponding residues in the inactive structures. Moreover, several residues acquire centrality when the structures are analyzed in the presence of ligands. Strikingly, there is little overlap between the residues that acquire centrality in the presence of the ligand in the blocker-bound structures and the agonist-bound structures.

**Conclusions:**

Our results reflect the fact that the receptor resembles a bow tie, with a rather tight knot of closely interconnected residues and two ends that fan out in two opposite directions: one toward the extracellular space, which hosts the ligand binding cavity, and one toward the cytosol, which hosts the G protein binding cavity. Moreover, they underscore how interaction network is by the conformational rearrangements concomitant with the activation of the receptor and by the presence of agonists or blockers.

**Electronic supplementary material:**

The online version of this article (doi:10.1186/2193-9616-1-16) contains supplementary material, which is available to authorized users.

## Background

G protein-coupled receptors (GPCRs) are a large group of integral membrane proteins of paramount pharmaceutical interest (Overington et al. 2006). Topologically, they feature a single polypeptide chain that spans the plasma membrane seven times, with seven α-helical transmembrane domains (TMs, numbered from TM1 to TM7). The N-terminus is in the extracellular milieu, the C-terminus is in the cytosol, and the seven TMs are connected by three extracellular loops (ELs, numbered from EL1 to EL3) and three intracellular loops (ILs, numbered from IL1 to IL3). In recent years, the field of GPCR crystallography has experienced a rapid expansion (Costanzi et al. 2009; Hanson and Stevens 2009; Stevens et al. 2013; Rosenbaum et al. 2009; Shukla et al. 2008; Lefkowitz et al. 2008), which is currently fostering the structure-based discovery of novel ligands for the receptors endowed with experimentally elucidated structures (Costanzi 2011; Mason et al. 2012; Jacobson and Costanzi 2012). Moreover, the entire superfamily of GPCRs is benefiting from this increase in structural knowledge, since the experimentally solved structures can serve as templates for the construction of computational models of those receptors that have yet to be solved experimentally (Costanzi 2010, 2012, 2013; Costanzi 2011; Costanzi and Wang 2014). This boom of GPCR crystallography led to the publication of several computational analyses intended to elucidate the molecular architecture of the receptors and/or probe the applicability of the three-dimensional structures to drug discovery (Jacobson and Costanzi 2012; Katritch et al. 2012, 2013; Venkatakrishnan et al. 2013; Mason et al. 2012).

In light of the wealth of information that surrounds it, we elected the β_2_ adrenergic receptor (β_2_ AR) as a model system for many of our computational studies of the GPCR superfamily (Costanzi and Vilar 2012; Vilar et al. 2011b; Vilar et al. 2011a; Vilar et al. 2010; Pooput et al. 2009). The (β_2_ AR) is a GPCR naturally activated by epinephrine and targeted by FDA approved drugs for a variety of indications (Pierce et al. 2002). In particular, blockers of the β_2_ AR are primarily employed as drugs for the treatment of hypertension, while agonists of the same receptor are chiefly used for the treatment of asthma (Johnson and Liggett 2011; Tashkin and Fabbri 2010). Among all GPCRs, the β_2_ AR is one of the most studied and better understood members of the superfamily, and is also the one for which the widest variety of experimentally elucidated structures have been solved (Kobilka 2011; Shukla et al. 2008; Lefkowitz et al. 2008). In particular, a total of 12 structures of the β_2_ AR were solved in complex with 7 different ligands (Rasmussen et al. 2007; Cherezov et al. 2007; Rosenbaum et al. 2007; Hanson et al. 2008; Bokoch et al. 2010; Wacker et al. 2010; Rosenbaum et al. 2011; Rasmussen et al. 2011a; Rasmussen et al. 2011b). Although most of the structures have been solved in an inactive state, two of the crystal structures of the β_2_ AR have been solved in a fully activated state, one in complex with a G protein-heterotrimer (PDB ID: 3SN6) and one in complex with a nanobody that mimics the G protein (PDB ID: 3P0G) (Rasmussen et al. 2011a; Rasmussen et al. 2011b). Of note, the 2012 Nobel Prize in Chemistry was awarded to Robert Lefkowitz and Brian Kobilka for their pioneering and fundamental contributions to the current understanding of the structure and function of the β_2_ AR (Lefkowitz 2013; Kobilka 2013).

**Figure 1:**
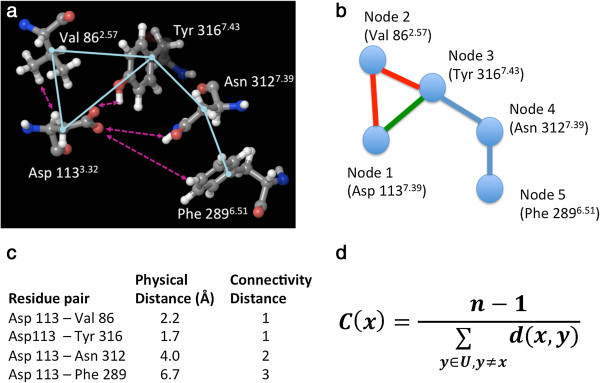
**Five interconnected residues in the carazolol-bound structure of the β**_**2**_ **AR (PDB ID: 2RH1).** Panel **a**: a three-dimensional atomic model of the five residues, with dotted double headed arrows indicating the shortest physical distance between Asp 113 and its neighboring residues and cyan lines indicating residues that establish contacts according to the CSU analysis; the residues are shown in balls and sticks format, with carbon atoms colored in gray, oxygen atoms in red, nitrogen atoms in blue and hydrogen atoms in white. Panel **b**: a graph representation in which the five residues are shown as nodes, with edges connecting the residues that establish contacts according to the CSU analysis. Panel **c**: physical distance between the closest atoms of Asp 113 and each other shown residue (represented as dotted double headed arrows in panel **a**) and connectivity distance between Asp 113 and each of the other residues shown, as inferred from the graph. All connectivity distances are calculated along the shortest paths. For instance, the green edge in panel a shows the shortest path from node 1 to node 3, while the red edges show an alternative longer path. Panel **d**: mathematical formula for the calculation of closeness-centrality (*C*) of node *x*, where *n* = number of nodes in the graph; *d(x, y)* = geodesic connectivity distance, i.e. the shortest path, between node *x* and node *y*; *U* = the set of all nodes. If the four shown residues were the only four nodes in the graph representation of the 2RH1 structure, their closeness centrality would be 0.57 for Val 86, 0.57 for Asp 113, 0.44 for Phe 289, 0.67 for Asn 312 and 0.80 for Tyr 316.

Here, we analyze the structure of the β_2_ AR through graph theory, a technique that has recently emerged as a tool applicable to the study of the global structural aspects of proteins (Di Paola et al. 2013; Amitai et al. 2004; Thibert et al. 2005; Tang et al. 2008; Slama et al. 2008; Pathak et al. 2013; del Sol et al. 2006a; Chea and Livesay 2007; del Sol et al. 2006b). In this approach, protein structures are described as a graph consisting of a number of nodes, *i.e.* the amino acid residues that make up the protein, connected by edges, *i.e.* the non-covalent interactions occurring between the residues (Figure 1a-c). Just like members of a social network, each residue of a protein has a certain number of first-degree connections, i.e. residues in direct contact, a certain number of second-degree connections, i.e. residues that are not in direct contact but share a common residue with which they are both in contact. Ultimately, each residue of the receptor is connected with every other residue of the receptor, although with different degrees of connectivity. This network of interconnected residues can be studied through the same analysis tools employed to analyze social networks of interconnected people, revealing patterns of connectivity, influential members, and dynamic behavior. Notably, in a seminal study published in 2006, Nussinov and coworkers applied graph theory to the study of rhodopsin, when this was still the only GPCR with an experimentally elucidated three-dimensional structure (del Sol et al. 2006b). In particular, the authors represented the structure of rhodopsin as a network of interacting residues and, removing residues from the network, identified those that play key roles in maintaining long-range interactions between distal regions of the receptor.

In this work, we focused on the analysis of the centrality of the various residues that compose the β_2_ AR, identifying the residues endowed with the highest centrality, comparing how the centrality of the same residues varies in structures that were solved in the inactive or the activated state, and studying the changes in centrality when the same structure was analyzed in the presence or the absence of the co-crystallized ligand.

The centrality of a member of a network is a measure of the extent to which the member in question is connected with other members of the network. There are several metrics to measure the centrality of the members of a network. Here, we specifically studied *closeness-centrality*, which is defined as the overall connectivity distance of the member in question (a node in the graph that represents the network) to all other members of the network (all other nodes in the graph). Mathematically, the closeness-centrality (*C*) of node *x* can be derived through the formula shown in Figure 1d, and results in a positive number that can assume the maximum value of 1, for nodes directly connected with every other node in the graph (Amitai et al. 2004).

**Figure 2:**
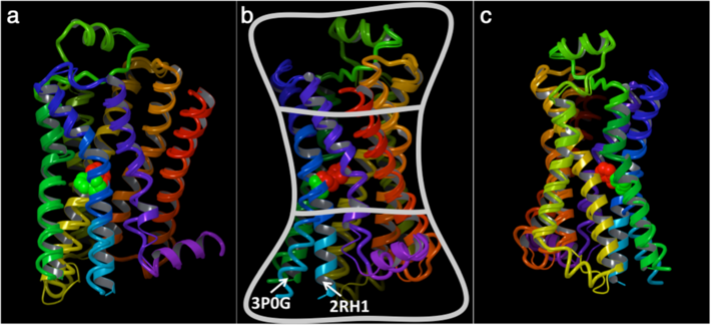
**Three alternative views of two of the experimentally solved structures of the β**_**2**_ **AR, one solved in the inactive state (2RH1) and one in an activated state (3P0G).** A key residue in the activation of the receptor, namely Phe 282^6.44^, is shown with its non-hydrogen atoms represented as spheres and colored in red for the 2RH1 structure and green for the 3P0G structure. The backbone of the receptor is schematically portrayed as a ribbon for the 2RH1 structure and as a tube for the 3P0G structure and is colored with a continuous spectrum ranging from red at the N-terminus to purple at the C-terminus, with TM1 in red/orange, TM2 in orange, TM3 in yellow, TM4 in yellow/green, TM5 in green, TM6 in blue and TM7 in purple. In the views provided in panels **b** and **c** the receptor is rotated clockwise (when observed from the extracellular space) of 60° and 340° degrees around its main axis with the respect to the view provided in panel **a**. The bow tie shape of the receptor is particularly evident from panel **b**.

In social networks, highly connected individuals tend to have a high influence on society (Wasserman and Faust 1994). Equally, residues endowed with high closeness-centrality have been found to be critical for the function of enzymes and to be generally located in the catalytic sites (Amitai et al. 2004; Thibert et al. 2005; Tang et al. 2008; Slama et al. 2008; Pathak et al. 2013; del Sol et al. 2006a; Chea and Livesay 2007). Notably, the same functional importance has been detected for nucleotide residues in ribosomes (David-Eden and Mandel-Gutfreund 2008). A further study from Nussinov and coworkers revealed that, for non-enzyme proteins, although residues endowed with high closeness-centrality are generally important for fold and function, they are often not located within their binding sites (del Sol et al. 2006a). The results of our study are completely in line with these findings. In particular, they revealed that the β_2_ AR residues endowed with the highest closeness-centrality are located in the core of the receptor. As a consequence, the receptor can be likened to a bow tie (Figure 2), with a rather tight knot of highly central and closely interacting residues and two ends that fan out in two opposite directions: one toward the extracellular space, which hosts the ligand binding cavity, and one toward the cytosol, which hosts the G protein binding cavity.

On a technical note, to transform each of several experimentally solved structures of the β_2_ AR into a graph representing a network of non-covalently connected residues, we utilized the CSU program, written by Sobolev and coworkers (Sobolev et al. 1999), to detect all non-covalent interactions present in each structure. Subsequently, we analyzed the closeness-centrality of each residue is in the context of the entire network of interactions by detecting the connectivity distance between each residue of the receptor with every other residue. This was done through an in-house implemented program based on an established algorithm for the identification of the shortest path that connects two nodes in a graph (Dijkstra 1959). As a clarification, the connectivity distance is not the actual physical distance between two residues, but is defined as the number of first degree connections (edges between the nodes) that separate two residues (Figure 1c) – hence, two directly connected residues will have a connectivity distance of 1, two residues that are not directly connected but share a common directly connected residue will have a connectivity of 2, and so on and so forth.

## Methods

### Inferring non-covalent interactions

The identification of non-covalent interactions was performed using the CSU program, which identifies contacting atoms based on excluded solvent accessible surface upon contact formation. (see (Sobolev et al. 1999) for the exact definition).

The source code for CSU was obtained from the Weizmann Institute’s website (http://ligin.weizmann.ac.il/space/programs/). To compile CSU, which is written in Fortran, we used a 64-bit Ubuntu 10.04 virtual machine hosted on the Amazon’s Elastic Compute Cloud (EC2) platform. The output data from CSU was processed using a custom-built wrapper script, written in the Python scripting language. This script facilitated the analysis by automatically identifying the number of chains present in the processed protein and running CSU on all pairs in a combinatorial manner. Furthermore, it parsed the output of CSU interaction into a hashmap, which encapsulated all the data relative to that interaction – The example shown in Additional file 1: Figure S1 shows how a CSU output line relative to a specific interaction would be transferred into the a Python dictionary. Then, filters were applied to the data to screen out any intra-residue interactions (e.g. interactions within atoms of the same residue), as well as covalent interactions between the backbone atoms of neighboring residues. The resulting data represented only non-covalent interactions between non-adjacent atoms.

### Construction of a graph describing the network of residue interactions and calculation of closeness-centrality

From the list of all non-convalent interactions between non-adjacent atoms, through another custom-built Python module, we constructed a graph representing all residue-residue interactions found in the protein.

First, the atomic interactions were organized in the form of residue-residue interactions. In particular, the interaction between a given source residue (denoted as Residue1) and a given destination residue (denoted as Residue2) was coded in the form a hashmap mapping the two residues onto the list of all the atomic interactions found between them, as the example in panel d of Additional file1: Figure S1 shows. A residue-residue interaction was always counted as one direct connection, despite the number of atomic interactions found between the two interacting residues.

Then, to determine the closeness-centrality, the connectivity distance from that residue (a specific node in the graph) to each other residue (each other node in the graph) following the shortest path was determined through Dijkstra’s algorithm (Dijkstra 1959). As explained in the introduction, the term connectivity distance is defined as the number of edges between the source and the destination node, not the actual physical distance between the nodes. For example, two nodes that share one direct interaction will have a connectivity distance of 1, while two nodes that do not directly interact but share one or more interactions with a common node will have a connectivity distance of 2. Dijkstra’s algorithm takes a specific node and computes the shortest path length to all other nodes in the graph by using a technique called relaxation. Initially, the connectivity distance from the source node to all other nodes is set as infinite. Then, the graph is traversed starting from the source. As new nodes are discovered in the process of traversing, the shortest path values become updated. The process continues until every node has been traversed, and the values presented are the shortest paths.

Once the shortest value connectivity distances from the source were obtained, the closeness-centrality of the source residue was determined according to the equation shown in Figure 1d. This process was repeated for every residue.

### GPCR residue identifier numbers

Throughout the paper, we referred to the β_2_ AR residues through their sequence number followed by a superscript indicating their GPCR residue identifier number. At this purpose, GPCR residue identifier numbers for all the β_2_ AR residues were calculated through a custom-built Python module that annotated the list of closeness-centrality data. Originally devised by Ballesteros and Weinstein, this system provides a universal way of identifying corresponding residues found in the 7 TMs of all GPCRs by numbering. In particular, this objective is achieved by assigning the identifier X.50, where X is the TM number, to the residue found in a reference alignment position chosen by the authors on the basis of sequence identity consideration. All other residues in the same TM domain are numbered relatively to the reference position. In the β_2_ AR, the X.50 positions are the following: 1.50, Asn 51; 2.50, Asp 79; 3.50, Arg 131; 4.50, Trp 158; 5.50, Pro 211; 6.50, Pro 288; and 7.50, Pro 323 (Ballesteros and Weinstein 1995).

### Addition of hydrogen atoms

**Figure 3:**
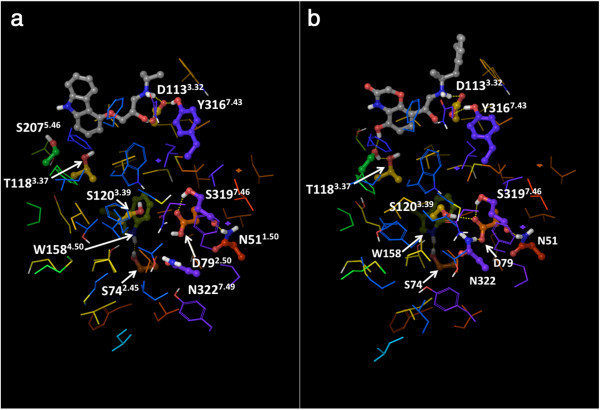
**Interhelical hydrogen bonds within the “knot region” of the β**_**2**_ **AR: a) The carazolol-bound inactive structure (2RH1); b) the active structure (3P0G).** The residues that form interhelical hydrogen bonds are shown as balls and sticks, all the others are shown as thin tubes. The color of the carbon atoms reflects the sequence position of the residues and goes from red at the N-terminus to purple at the C-terminus, with TM1 in red/orange, TM2 in orange, TM3 in yellow, TM4 in yellow/green, TM5 in green, TM6 in blue and TM7 in purple. The ligands co-crystallized with the receptors are also shown as balls and sticks, with their carbon atoms colored in gray. Hydrogen bonds are represented as yellow dotted lines.

**Figure 4:**
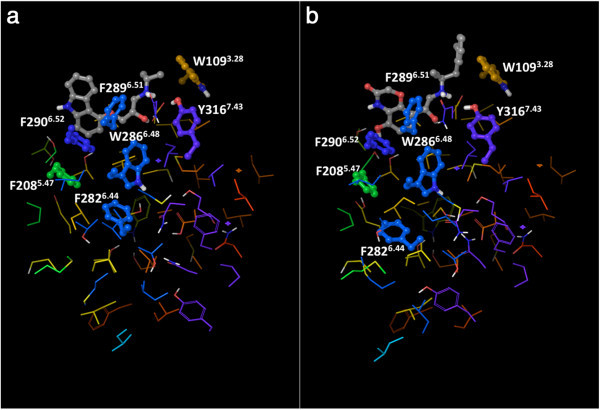
**View of the “knot region” of the β**_**2**_ **AR, with the cluster of interconnected aromatic residues highlighted: a) the carazolol-bound inactive structure (2RH1); b) the active structure (3P0G).** The residues that belong to the aromatic cluster are shown as balls and sticks, all the others are shown as thin tubes. The color of the carbon atoms reflects the sequence position of the residues and goes from red at the N-terminus to purple at the C-terminus, with TM1 in red/orange, TM2 in orange, TM3 in yellow, TM4 in yellow/green, TM5 in green, TM6 in blue and TM7 in purple. The ligands co-crystallized with the receptors are also shown as balls and sticks, with their carbon atoms colored in gray.

**Figure 5:**
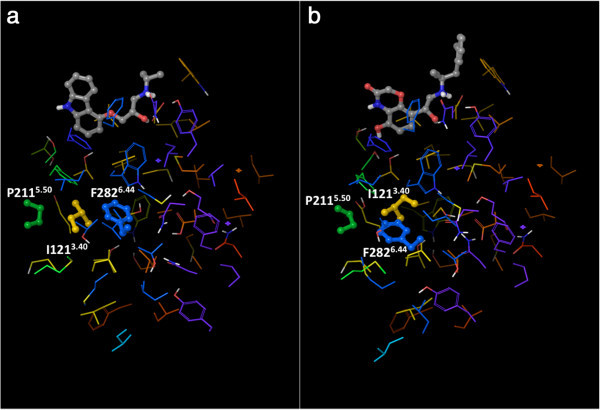
**View of the “knot region” of the β**_**2**_ **AR, with the P-I-F motif highlighted: a) The carazolol-bound inactive structure (2RH1); b) the active structure (3P0G).** The residues that belong to the P-I-F motif are shown as balls and sticks, all the others are shown as thin tubes. The color of the carbon atoms reflects the sequence position of the residues and goes from red at the N-terminus to purple at the C-terminus, with TM1 in red/orange, TM2 in orange, TM3 in yellow, TM4 in yellow/green, TM5 in green, TM6 in blue and TM7 in purple. The ligands co-crystallized with the receptors are also shown as balls and sticks, with their carbon atoms colored in gray.

The identification of the non-covalent interactions did not require the addition of hydrogen atoms to the crystal structures, since CSU has been expressly designed to implicitly account their presence. However, for the preparation of Figures 1, 3, 4 and 5, we added hydrogen atoms with the “protein preparation wizard” workflow of the Schrödinger suite, to add hydrogen atoms and calculate the protonation states of ionizable groups at pH 7. The workflow also optimized the orientation of hydroxyl groups, as well as Asn, Gln and His residues (Schrödinger Suite 2012; Sastry et al. 2013).

### Preparation of the figures

All the figures representing three-dimensional structures of proteins were prepared with the Schrödinger suite (Schrödinger Suite 2012).

## Results

**Figure 6:**
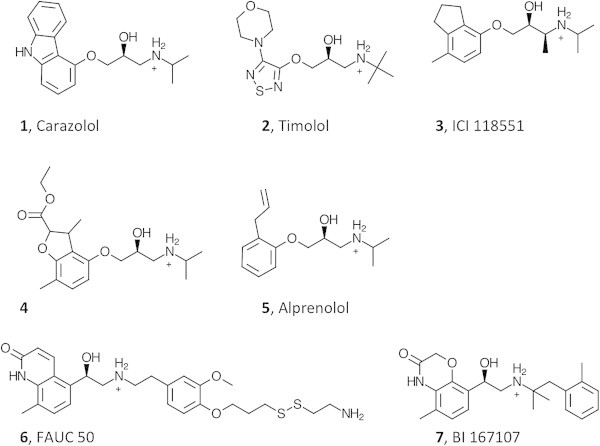
**Molecular structures of the seven ligands crystallized with the β**_**2**_ **AR in the published structures.** Compounds **1–** **5** are blockers, while compound **6–7** are agonists. Of note, compound **6** is covalently bound to a Cys residue artificially introduced in place of His 93^2.64^ in TM2.

A total of 12 crystal structures have been solved for the β_2_ AR, in complex with 7 different ligands (Figure 6). Our study is based on the analysis of 7 of these structures, each of which is representative of the complex between the receptor and one of the 7 different ligands with which it has been co-crystallized. In order to make the comparison among different structures possible, we analyzed only the portion of the receptor solved in all the studied structures, expunging the residues that were solved only in a subset of the structure – the resulting amino acid sequence of the analyzed structures is shown in Additional file 1: Figure S2. Specifically, the analyzed sequence comprised the entire segment (including all the intervening loops) from the beginning of TM1 to the end of TM4 and the entire segment (including all the intervening loops) from the beginning of TM5 to the end of helix 8 (H8), which is an amphipathic cytosolic helix that immediately follows TM7 (Additional file 1: Figure S2).

In particular, the analyzed structures, identified through their PDB ID, with their respective resolution indicated in parentheses, are: a) 2RH1 (2.40 Å) (Cherezov et al. 2007; Rosenbaum et al. 2007), 3D4S (2.80 Å) (Hanson et al. 2008), 3NY8 (2.84 Å) (Wacker et al. 2010), 3NY9 (2.84 Å) (Wacker et al. 2010), and 3NYA (3.16 Å) (Wacker et al. 2010), which were solved in the inactive state in complex with the blockers carazolol (1), timolol (2), ICI 118551 (3), a blocker recently identifies through virtual screening (4) (Kolb et al. 2009), and alprenolol (5), respectively; b) 3PDS (3.50 Å) (Rosenbaum et al. 2011), which was solved in the inactive state in complex with the agonist FAUC 50, a large compound covalently bound to a Cys residue artificially introduced in place of His 93^2.64^ in TM2 (6); c) 3P0G (3.50 Å) (Rasmussen et al. 2011a), which was solved in complex with the agonist BI 167107 (7) and the nanobody nb80. Between the two activated structures of the receptor solved in complex with the agonist BI 167107, we chose 3P0G rather than 3SN6, although the latter features the presence of a heterotrimeric G protein. This choice was dictated by the fact the side-chains of several amino acids that were solved for all the seven analyzed structures, were not solved for 3SN6.

For brevity, throughout the Results and Discussion sections of this article, we refer to the closeness-centrality, defined by the formula in Figure 1d, simply as centrality. Moreover, we identify all amino acid residues through both their sequence number and their GPCR index number – see methods for more information.

### The most connected residues

**Figure 7:**
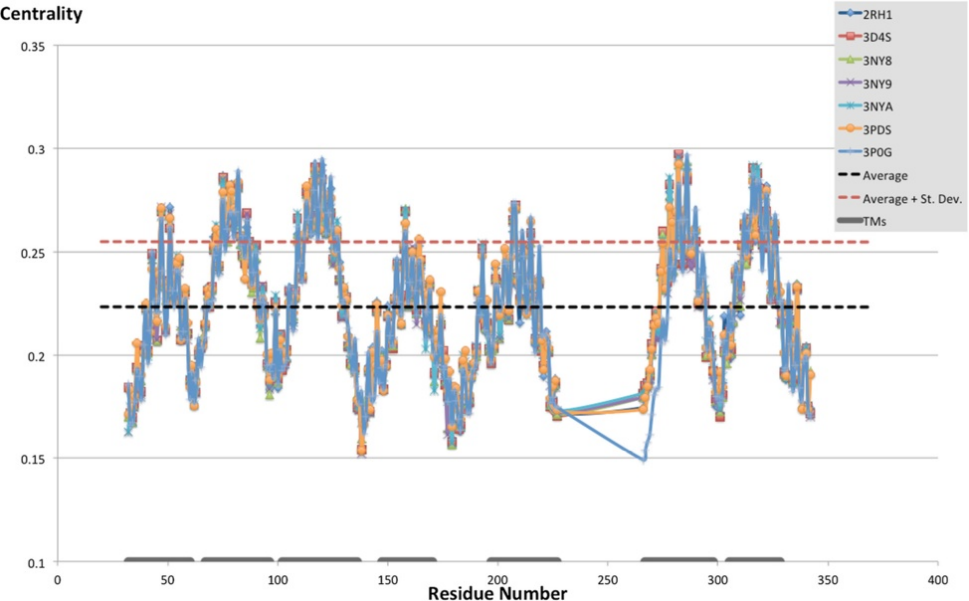
**Closeness-centrality of all residues in the seven analyzed structures.** The seven peaks of high closeness-centrality identify residues located in the middle of each of the seven transmembrane domains of the receptor. In particular, most of the residues characterized by high closeness-centrality are located in the middle of TM2, TM3, TM6 and TM7.

**Figure 8:**
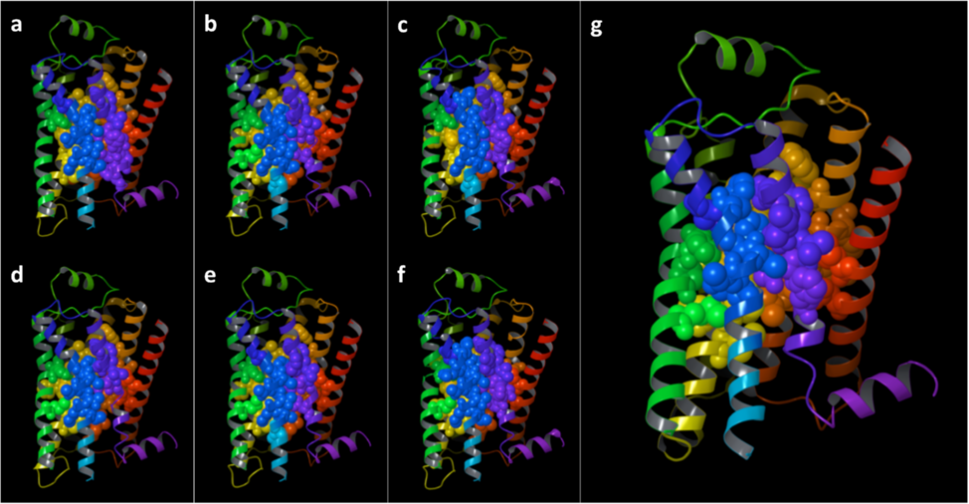
**Three-dimensional representation of the β**_**2**_ **AR, showing the residues for which the detected closeness-centrality exceeded the average plus the standard deviation of the values detected across all residues for the 2RH1 (panel a), 34DS (panel b), 3NY8 (panel c), 3NY9 (panel d), 3NYA (panel e), 3PDS (panel f), and 3P0G (panel g) structures.** The backbone of the receptor is schematically portrayed as a ribbon colored with a continuous spectrum ranging from red at the N-terminus to purple at the C-terminus, with TM1 in red/orange, TM2 in orange, TM3 in yellow, TM4 in yellow/green, TM5 in green, TM6 in blue and TM7 in purple. The non-hydrogen atoms of the shown residues are represented as spheres, colored according to the same scheme illustrated for the backbone.

Our first level of analysis focused on the identification of the residues with the highest centrality in the seven studied structures. As Figure 7 shows, plotting the centrality value versus the residue numbers reveals the presence of seven peaks corresponding to regions with highly connected residues. Mapping on the crystal structures the most highly connected residues – more specifically the residues for which the centrality value was equal or higher than a cutoff value set to the average plus the standard deviation across all residues (centrality ≥ 0.255) – reveals that each of these seven regions is located in the middle of one of the seven transmembrane domains of the receptor (Figure 8). The centrality values for the mapped residues are numerically reported in Additional file 1: Table S1, which reveals that most of the highly connected residues are located in the middle of TM2, TM3, TM6 or TM7, while a substantially narrower number of highly connected residues are located in the middle of TM1, TM4 or TM5. There are no substantial differences in the pool of residues with the highest centrality in the seven analyzed structures. The only notable exception concerns the activated structure (3P0G), for which residues located toward the cytosolic end of the middle portion of TM6 show a lower centrality when compared to their counterparts in the inactive structures.

### Differences in centrality between activated and inactive structures

**Figure 9:**
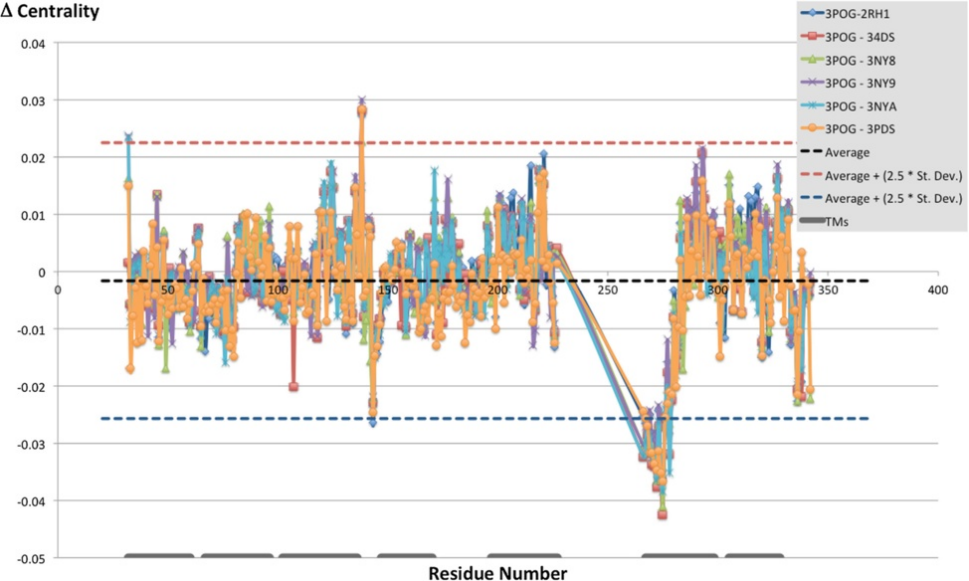
**Difference in closeness-centrality between the activated structure (3POG) and the rest of the analyzed structures, which are all inactive: TM6 looses centrality with activation.**

**Figure 10:**
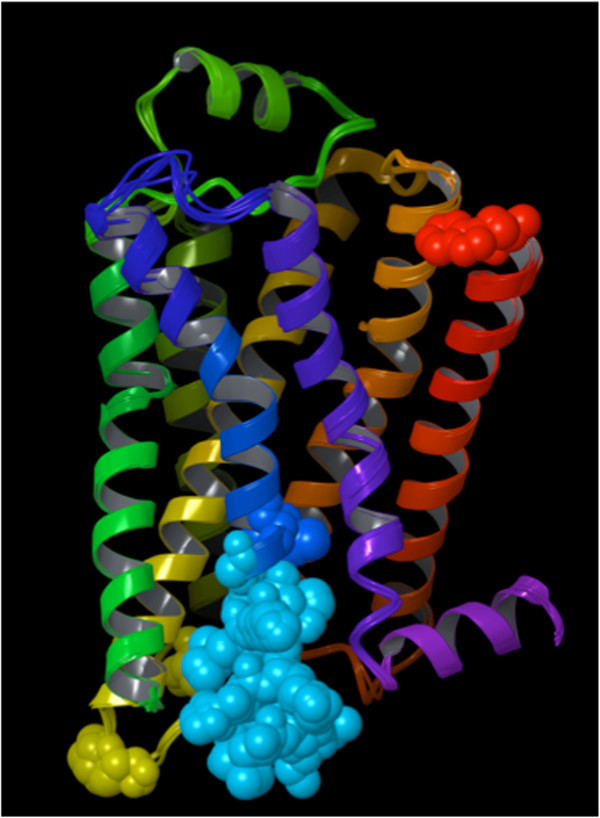
**Three-dimensional representation of the β**_**2**_ **AR, showing the residues for which the difference in closeness-centrality between the activated structure (3POG) and the rest of the analyzed structures exceeded the average plus 2.5 times the standard deviation of the values detected across all residues – separate figures for the individual structures are given in Additional file** **1** **: Figure S3.** The backbone of the receptor is schematically portrayed as a ribbon colored with a continuous spectrum ranging from red at the N-terminus to purple at the C-terminus, with TM1 in red/orange, TM2 in orange, TM3 in yellow, TM4 in yellow/green, TM5 in green, TM6 in blue and TM7 in purple. The non-hydrogen atoms of the shown residues are represented as spheres, colored according to the same scheme illustrated for the backbone.

Our second level of analysis delved more deeply into the study of the difference in centrality among the seven structures. In particular, for each residue, we studied the difference between the centrality detected for the activated structure (3P0G) and each of the six inactive structures. As the plot shown in Figure 9 reveals, there is one region for which the centrality detected for the active structure is markedly different from that of the corresponding residues in the inactive structure. Mapping on the crystal structures the residues with the most marked differences in centrality – more specifically the residues for which the difference in centrality exceeded cutoff values set to the average plus or minus 2.5 times the standard deviation across all residues (difference in centrality ≥ 0.022 or ≤ −0.026) – reveals that the region in which most of the differences are concentrated is located toward the cytosolic end of TM6 (Figure 10 and Additional file 1: Figure S3). More specifically, residues located in this region show a higher centrality in the inactive structures than in the activated structures. A further region for which differences in centrality were detected is the second intracellular loop, with one residue that features a higher centrality in the inactive than the activated structure (Pro 138) and one that acquires centrality with the activation (Ser 143). Of note, this domain was crystallized in an unstructured conformation in the inactive structures and as a α-helix in the activated structures (see panel c of Figure 2). Moreover the residue located at the extracellular end of TM1 (Trp 32^1.33^), features a marked lower centrality in two of the analyzed structures, namely 3NY9 and 3NYA, than the rest of the analyzed structures – it is worth noting, however, that for the 3NY9 structure the side chain of Trp 32^1.33^ was not solved. The numerical values of the difference in centrality values for the mapped residues are numerically reported in Additional file 1: Table S2.

### Effect of the co-crystallized ligands on the centrality of the β_2_ AR residues

**Figure 11:**
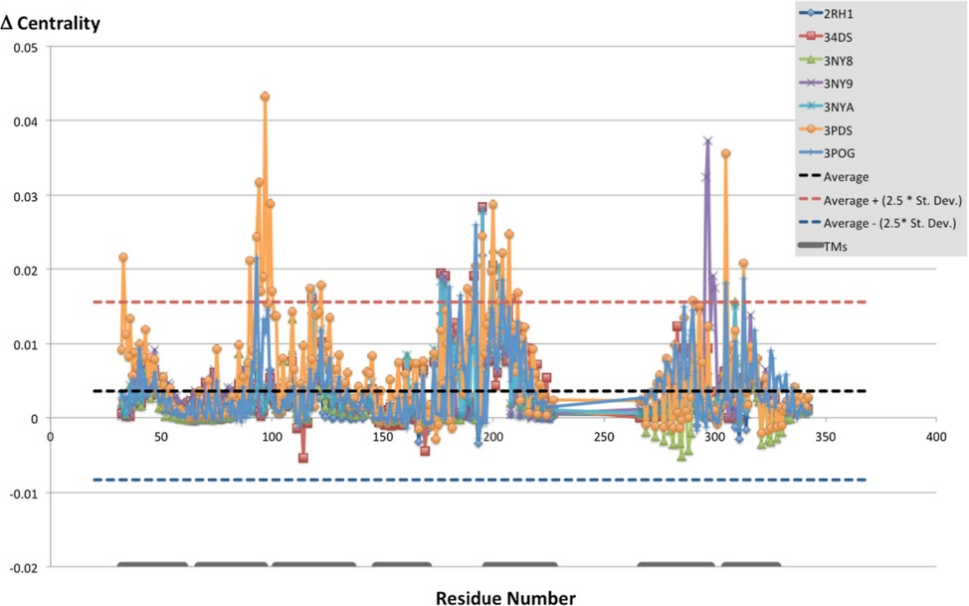
**Change in closeness-centrality detected when the structures are analyzed with or without ligands.** With the exception of Cys 191 and Ser 204, there is complete lack of overlap between the residues that acquire centrality in the blocker-bound structures and those that acquire centrality in the activated agonist-bound structure (3P0G).

**Figure 12:**
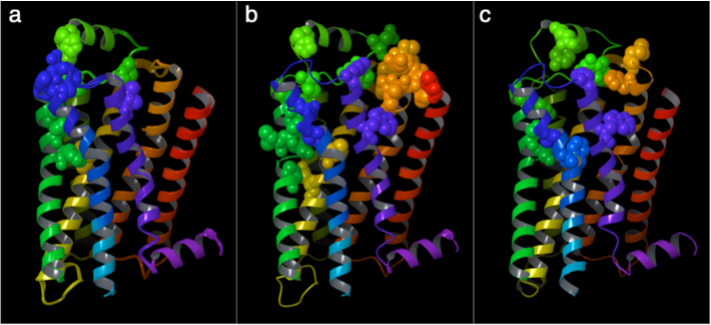
**Three-dimensional representation of the β**_**2**_ **AR, showing the residues for which the difference in closeness-centrality detected when the analyses were performed in the presence or the absence of ligands exceeded the average plus 2.5 times the standard deviation of the values detected across all residues, for all the analyzed blocker-bound structures (panel a), as well as the agonist-bound 3PDS (panel b) and 3POG (panel c). Separate figures for the individual blocker-bound structures are given in Additional file** **1** **: Figure S4.** The backbone of the receptor is schematically portrayed as a ribbon colored with a continuous spectrum ranging from red at the N-terminus to purple at the C-terminus, with TM1 in red/orange, TM2 in orange, TM3 in yellow, TM4 in yellow/green, TM5 in green, TM6 in blue and TM7 in purple. The non-hydrogen atoms of the shown residues are represented as spheres, colored according to the same scheme illustrated for the backbone.

In the first level of analysis, the structures were analyzed without the co-crystallized ligands. Thus, in our second level of analysis we studied the change in centrality detected when the structures were analyzed with or without the co-crystallized ligand. Because the ligands in question are small molecules, we treated them as a single residue. As the plot shown in Figure 11 reveals, there is one major region for which the centrality is markedly increased in the presence of the ligands. Mapping on the crystal structures the residues most markedly affected by the ligands – more specifically the residues for which the difference in centrality was equal or higher than a cutoff value set to the average plus 2.5 times the standard deviation across all residues (difference in centrality ≥ 0.015) – reveals that the region in question comprises residues in EL2 and the adjacent half of TM5 (Figure 12 and Additional file 1: Figure S4). A closer examination of Figure 12 and Additional file 1: Table S3 reveals that additional regions that acquire centrality for the blocker-bound structures include TM3, TM6, TM7 and EL3, while additional regions that acquire centrality for the agonist-bound structures include TM1, TM2, TM3, TM6, TM7 and EL1. Despite this partial overlap, the pattern of residues that acquire centrality in the presence of agonists or blockers is rather different. Strikingly, there is little overlap between the residues that acquire centrality in the presence of the ligand in the blocker-bound structures and the agonist-bound structures. In particular, only two residues are in common between the two agonist-bound structures and some of the blocker-bound structures, namely Cys 191^EL2^ and Ser 204^5.43^. Moreover, the inactive structure crystallized with the large, covalently bound, FAUC 50 agonist (3PDS) shares two additional residues in common with some of the blocker-bounds structures, namely Ala 200^5.39^, Ser 203^5.42^ (Additional file 1: Table S3).

## Discussion

As mentioned, following the recent boom of GPCR crystallography, several analyses of the molecular architecture of the receptors were published (Jacobson and Costanzi 2012; Katritch et al. 2012, 2013; Venkatakrishnan et al. 2013; Mason et al. 2012). These studies focused on the analysis of the individual residue-residue and residue-ligand interactions found in the now numerous experimentally elucidated structures of GPCRs. Even prior to the flourishing of the field of GPCR structural biology, through sequence alignments and homology modeling, a number of studies reported hypotheses on the putative individual interactions that kept together the structure of the receptors and facilitated the interactions with their ligands. Some of these studies were extended to the entire GPCR superfamily (Ballesteros et al. 2001), while others, such as those in the examples shown in the cited references, were confined to the analysis of single families of receptors (Costanzi et al. 2004; Kim et al. 2003). The present analysis of the β_2_ AR complements these studies by adding a description of the ability of each residue of this prototypical GPCR to establish direct or indirect relationships with all other residues of the receptor.

Our study highlighted that the receptor resembles a bow tie, with a rather tight knot in correspondence of the region of the helical bundle deeply buried in the middle of the plasma membrane, where the most central residues are located (Figure 2). It is in the correspondence of this knot that the most highly connected residues are found. Of the seven transmembrane domains, those that most actively contribute to keeping the knot tight are the more internal TM2, TM3, TM6 and TM7 (Figure 6). A recent detailed analysis of the crystal structures of all GPCRs revealed the existence of a consensus scaffold of non-covalent residue-residue contacts between 36 topologically equivalent residues common to all the solved structures [(Venkatakrishnan et al. 2013)]. Notably, our analysis based on graph theory revealed that 25 out of these 36 topologically equivalent residues are residues endowed with high centrality in the knot region of the receptor.

From the knot, two ends characterized by lower centrality values fan out in two opposite directions, one toward the cytosol and one toward the extracellular milieu. These regions are endowed with lower centrality values and correspond to the two well characterized cavities found in GPCRs: as crystallographic evidence showed for β_2_ AR and rhodopsin, the cavity opened toward the cytosol hosts the site to which the C-terminus of the α subunit of the G protein binds, while the one opened the toward the extracellular milieu hosts the ligand binding site (Rasmussen et al. 2011b; Scheerer et al. 2008; Choe et al. 2011; Standfuss et al. 2011; Deupi et al. 2012; Singhal et al. 2013). In light of the cavities present within them and their more peripheral location, it is not surprising that, as mentioned, the two ends feature residues endowed with a substantially lower network centrality than the residue in the knot. This observation is in line with the finding of Nussinov and coworkers that, although for enzymes the residues endowed with the highest centrality typically line binding cavities, the same is not necessarily true for other proteins (del Sol et al. 2006a). Conversely, in a different study in which graph-theory was not applied to the analysis of structures but to the examination of an alignment of GPCR sequences, it was found that the residues that, for class A and class C receptors, share the highest mutual information are located in the cavity opened toward the extracellular milieu (Fatakia et al. 2009).

GPRS are inherently flexible molecules that exist in a continuum spectrum of conformations associated with different levels of activation and, most likely, responsible for the triggering of different signaling cascades (Kobilka and Deupi 2007; Yao et al. 2006; Swaminath et al. 2005; Swaminath et al. 2004; Kim et al. 2013; Nygaard et al. 2013; Bokoch et al. 2010). Hence, the network centrality of the residues that compose them changes as a consequence of the conformational rearrangements of the receptor. Specifically, in our study, when comparing the network of connections of the β_2_ AR residues in activated versus inactive structures, we observed a marked loss in centrality for the residues located in the cytosolic half of TM6 (Figure 10). This result is completely in line with what was eloquently illustrated by the crystal structure of the β_2_ AR in complex with a G_s_ heterotrimer as well as those of rhodopsin in complex with the C-terminal fragment of the α subunit of transducing [Refs]. According to these structures, the most obvious structural change consequent the activation of the receptor is an opening of the cytosolic portion of the helical, which is absolutely necessary for the formation of a docking cavity for the C-terminus of the G protein and occurs through a dramatic outward swing of the entire half of TM6 that faces the cytosol. In particular, the cytosolic end of TM6 moves away of about 14 Å from the space that it occupies when in the inactive conformation (Figure 2) (Rasmussen et al. 2011a).

The end of the bow tie that stems from the knot toward the extracellular milieu, as mentioned, harbors the ligand-binding cavity. As recently reviewed, in each receptor this cavity is endowed with a particular shape and size, ranging from the large and widely open cleft found in peptide-binding receptors to the tight and more enclosed site found in the muscarinic receptors (Jacobson and Costanzi 2012). As ligands bind to their receptors, they establish contacts with the residues of the ligand-binding cavity, therefore increasing their centrality. For the β_2_ AR, our analysis revealed that agonists and blockers contribute to enhancing the centrality of substantially different sets of residues (Figure 12). In all likelihood, the pattern of connectivity fostered by agonists is the cause of the stabilization of specific signaling states of the receptor. This is in line with the experimental observation that agonists and blockers of GPCRs typically show patterns of receptor-ligand interactions that are not completely overlapping, as the crystal structures and a number of detailed analyses highlighted (Rasmussen et al. 2011a; Deflorian et al. 2012; Xu et al. 2011; Jacobson and Costanzi 2012; Venkatakrishnan et al. 2013; Katritch et al. 2013).

Closeness-centrality values indicate the most connected residues. However, they do not reveal the details of the interactions established by the residues. Hence, following the analysis based on graph theory, we performed a visual analysis of the first-degree residue-residue connections for the residues found in the knot. In the remainder of the Discussion, we describe the main features highlighted by this analysis and examine a putative role of residues found in the knot in linking agonist binding to receptor activation that seem plausible in light of the available structural information.

Our visual analysis revealed that many of the first-degree contacts of the residues in the knot are established with other residues also found in the knot. We postulate that these first-degree connections among knot residues are likely responsible for maintaining the α-helical structure of the transmembrane domains and for keeping together the heptahelical bundle. Concerning their nature, our visual inspection revealed that only few of them are interhelical hydrogen bonds (Figure 3). Specifically, interhelical hydrogen bonds were detected between the pairs Asn 51^1.50^ – Ser 319^7.46^ (backbone), Ser 74^2.45^ – Trp 158^4.50^, Asp 79^2.50^– Ser 319^7.46^, and Asp 113^3.32^ – Tyr 316^7.43^ for all the analyzed structures. An additional hydrogen bond was detected between the pair Asp 79^2.50^ – Asn 322^7.49^ for both agonist-bound structures (3PDS and 3P0G), and was caused by a rearrangement of the side chain of Asn 322^7.49^ and the protonation state assigned to Asp 79^2.50^. Of note, in agreement with what was suggested in the literature (Ghanouni et al. 2000; Vanni et al. 2010), Asp 79^2.50^ was predicted to be protonated for the inactive structures and deprotonated for the agonist-bound structures by the hydrogen-bond network optimization tool that we employed. Moreover, two additional hydrogen bonds were detected between the pairs Asp 79^2.50^ – Ser 120^3.39^, Ser 207^5.46^ – Thr118^3.37^ for the agonist-bound activated structure only (3P0G), which are due to the considerable rearrangements that Ser 120^3.39^ and Ser 207^5.46^ undergo upon activation.

The other contacts among the residues in the knot typically involve hydrophobic atoms. For instance, aromatic contacts interconnect a cluster of aromatic residues that comprises Trp 109^3.28^, Phe 208^5.47^, Phe 282^6.44^, Phe 282^6.44^, Trp 286^6.48^, Phe 289^6.51^, Phe 290^6.52^, and Tyr 316^7.43^ (Figure 4). Moreover, hydrophobic contacts are also found in the case of three notable interacting residues dubbed the “P-I-F” motif, namely, Pro 211^5.50^, Ile 121^3.40^ and Phe 282^6.44^ (Figure 5) – note that the latter residue also belongs the above-mentioned aromatic cluster. Residues of the P-I-F are conserved in many aminergic receptors and connect TM3, TM5 and TM6 (Rasmussen et al. 2011a; Wacker et al. 2013). As Kobilka and coworkers demonstrated, in the agonist-bound fully activated structure of the receptor, Phe 282^6.44^ of the P-I-F motif shows a major conformational change with respect to the blocker-bound inactive structures (Figure 2) (Rasmussen et al. 2011a). Experimental evidence shows that mutation of Phe 282^6.44^ leads to a receptor with higher increased or decreased basal activity, depending on the substituting amino acid (Chen et al. 2002). Hence, one can speculate that this event could be the likely trigger of the outward swing of the entire portion of TM6 upstream of the phenylalanine, i.e. the entire half of TM6 that faces the cytosol.

In the β_2_ AR, the P-I-F motif is not in direct contact with the ligand. Thus, it is reasonable to venture that some of the residues that acquire centrality upon agonist binding may provide a link between the agonist, the P-I-F motif and TM6. Two residues belonging to the above-mentioned aromatic cluster found in the knot region, namely Trp 286^6.48^ and Phe 290^6.52^, are among those that acquire connectivity in the presence of agonists but not blockers. Within this cluster, the aromatic residue at position 6.48, which is a staple of most class A GPCRs, is one of the most studied residues across the superfamily. For many receptors, although not all, mutations of this residue have been reported to significantly alter the activation process. For some GPCRs, for instance the purinergic P2Y_1_ receptor, its mutation to alanine leads to a receptor that can no longer be activated by agonists, despite their binding (Costanzi et al. 2004). For others, such as the TRH-R1 receptor, it leads to a constitutively active receptor (Deflorian et al. 2008; Sun and Gershengorn 2002). Finally, for others yet, such as the serotonin 5-HT_4_ receptor, it suppresses basal activity but does not alter the maximal activity of the natural agonist (Pellissier et al. 2009). From the structural point of view, on the basis of early biophysical experiments, this residue was hypothesized to undergo a substantial conformational change with the activation of the receptor and was dubbed the “rotamer toggle switch” (Shi et al. 2002; Yao et al. 2006). Among the GPCR structures that were solved in at least a partially activated state, this putative conformational change is most evident for the adenosine A_2A_ receptor, although perhaps not as dramatic as expected (Xu et al. 2011; Katritch et al. 2013). Conversely, for the β_2_ AR, the conformational change of Trp 286^6.48^ and the adjacent Phe 290^6.52^ captured through X-ray crystallography are subtler (Figure 4) (Rasmussen et al. 2011a; Rasmussen et al. 2011b). Hence, Trp 286^6.48^ and Phe 290^6.52^ may not be the primary links between agonists and the P-I-F motif.

Conversely, another residue that in our analysis acquires centrality only in the presence of agonists, namely Ser 207^5.46^, is likely to provide a stronger physical link between agonist binding and the conformational rearrangement of the P-I-F motif. As the comparison between active and inactive structures of the β_2_ AR clarified, the largest movement detected in the ligand binding cavity as a consequence of the activation of the receptor is a shift of the portion of TM5 centered around this serine residue, which moves 2.1 Å toward the center of the helical bundle, attracted by the aromatic hydroxyl groups of the bound agonists (Figure 3) (Rasmussen et al. 2011a; Rasmussen et al. 2011b). As hypothesized by Rasmussen and coworkers, this rearrangement of Ser 207^5.46^, which through a series of conformational searches coupled with statistical analyses we had predicted to be associated with the activation of the β_2_ AR prior to the solution of the activated structure (Vilar et al. 2011b), may trickle down to Pro 211^5.50^ of the P-I-F motif, located one helix turn below Ser 207^5.46^. In turn, this could be the cause of the consequent rearrangement of the two remaining residues of the motif, (Figure 5) (Rasmussen et al. 2011a), which is likely to be responsible for triggering the large outward swing of the portion of TM6 upstream of Phe 282^6.44^.

## Conclusions

In conclusion, our analysis of the structure of the β_2_ AR based on graph theory highlighted that the receptor resembles a bow tie, with a knot of closely interconnected residues and two ends that fan out in two opposite directions: one toward the extracellular space, which hosts the ligand binding cavity, and one toward the cytosol, which hosts the G protein binding cavity. Moreover, the results highlighted how the intricate network of interactions among the residues of the receptor is affected by the presence of agonists or blockers as well as the conformational rearrangements concomitant with the activation process.

## Electronic supplementary material

Additional file 1: Table S1: Residues showing the highest network centrality; **Table S2.** Difference in network closeness-centrality with respect to 3POG; **Table S3.** Change in network centrality when the structures are analyzed with or without ligands; **Figure S1.** Example showing how non-covalent interactions are stored in Python dictionary. **Figure S2.** Amino acid sequence of the β2 AR structures subjected to the analysis; **Figure S3.** The six individual structures that are shown superimposed in Figure 10; **Figure S4.** The five individual structures that are shown superimposed in panel a of Figure 12. (PDF 1 MB)
