# Supplementary material for: Graph analysis of β2 adrenergic receptor structures: a “social network” of GPCR residues
Source: In Silico Pharmacol. 2013 Dec 5;1:16. doi: 10.1186/2193-9616-1-16 (PMC4230308; doi:10.1186/2193-9616-1-16)

Supporting information to:

Graph analysis of β_2 adrenergic receptor structures: a dynamic "social Network" of GPCR residues

Samuel Sheftel,[a] Kathryn E. Muratore,[a] Michael Black,[b] and Stefano Costanzi[a,c,*]

[a]Department of Chemistry, [b]Department of Computer Science, and [c]Center for Behavioral Neuroscience, American University, Washington, DC 20016, USA

Table of Contents **Page**

Supplementary Table 1. 2
Supplementary Table 2. 4
Supplementary Table 3. 5
Supplementary Figure 1. 7
Supplementary Figure 2. 8
Supplementary Figure 3. 9
Supplementary Figure 4. 10

Supplementary Table 1. Residues for which the centrality value was higher than a cutoff value set to the average plus the standard deviation across all residues showing the highest network centrality (cutoff 0.255).

			Centrality [a]						
**Residue**	**Domain**	**GPCR ident.**	**2RH1**	**3D4S**	**3NY8**	**3NY9**	**3NYA**	**3PDS**	**3P0G**
Ile 47	TM1	1.46	0.271	0.270	0.270	0.272	0.267	0.271	0.266
Asn 51	TM1	1.50	0.272	0.261	0.268	0.268	0.262	0.266	0.262
Phe 71	TM2	2.42	0.257	0.251	0.252	0.252	0.251	0.253	0.248
Ile 72	TM2	2.43	0.261	0.257	0.260	0.257	0.263	0.261	0.252
Ser 74	TM2	2.45	0.260	0.260	0.260	0.260	0.259	0.259	0.254
Leu 75	TM2	2.46	0.287	0.286	0.285	0.285	0.284	0.279	0.275
Ala 76	TM2	2.47	0.261	0.261	0.258	0.261	0.266	0.261	0.250
Cys 77	TM2	2.48	0.265	0.264	0.255	0.256	0.262	0.264	0.261
Ala 78	TM2	2.49	0.274	0.279	0.281	0.271	0.280	0.282	0.269
Asp 79	TM2	2.50	0.282	0.281	0.273	0.281	0.276	0.279	0.269
Leu 80	TM2	2.51	0.263	0.266	0.263	0.265	0.263	0.271	0.256
Val 81	TM2	2.52	0.266	0.269	0.270	0.269	0.267	0.269	0.269
Met 82	TM2	2.53	0.281	0.282	0.281	0.281	0.281	0.284	0.289
Gly 83	TM2	2.54	0.257	0.257	0.253	0.257	0.254	0.258	0.253
Leu 84	TM2	2.55	0.249	0.248	0.248	0.255	0.249	0.254	0.258
Val 86	TM2	2.57	0.262	0.269	0.261	0.268	0.264	0.255	0.265
Trp 109	TM3	3.28	0.258	0.266	0.259	0.266	0.269	0.254	0.262
Ile 112	TM3	3.31	0.257	0.257	0.259	0.256	0.258	0.258	0.258
Asp 113	TM3	3.32	0.278	0.279	0.278	0.278	0.278	0.282	0.274
Val 114	TM3	3.33	0.269	0.270	0.271	0.270	0.271	0.272	0.265
Leu 115	TM3	3.34	0.258	0.264	0.266	0.270	0.261	0.262	0.258
Cys 116	TM3	3.35	0.276	0.283	0.286	0.275	0.282	0.284	0.280
Val 117	TM3	3.36	0.288	0.291	0.290	0.288	0.288	0.290	0.293
Thr 118	TM3	3.37	0.260	0.267	0.264	0.267	0.262	0.265	0.256
Ala 119	TM3	3.38	0.261	0.268	0.264	0.266	0.263	0.261	0.271
Ser 120	TM3	3.39	0.292	0.285	0.286	0.288	0.285	0.284	0.295
Ile 121	TM3	3.40	0.273	0.271	0.273	0.271	0.270	0.278	0.285
Glu 122	TM3	3.41	0.261	0.259	0.260	0.259	0.258	0.267	0.259
Thr 123	TM3	3.42	0.273	0.267	0.266	0.267	0.266	0.265	0.269
Leu 124	TM3	3.43	0.280	0.269	0.271	0.271	0.267	0.276	0.286
Cys 125	TM3	3.44	0.252	0.247	0.246	0.244	0.247	0.256	0.261

Ile 127	TM3	3.46	0.261	0.258	0.258	0.261	0.265	0.261	0.257
Trp 158	TM4	4.50	0.269	0.269	0.271	0.270	0.270	0.264	0.260
Thr 164	TM4	4.56	0.253	0.241	0.240	0.242	0.243	0.256	0.245
Ser 207	TM5	5.46	0.260	0.265	0.266	0.264	0.264	0.270	0.274
Phe 208	TM5	5.47	0.271	0.272	0.271	0.271	0.271	0.271	0.274
Pro 211	TM5	5.50	0.247	0.249	0.248	0.247	0.248	0.254	0.260
Met 215	TM5	5.54	0.248	0.259	0.254	0.257	0.259	0.265	0.267
Leu 275	TM6	6.37	0.253	0.260	0.258	0.253	0.256	0.254	0.217
Ile 278	TM6	6.40	0.279	0.282	0.277	0.274	0.286	0.271	0.250
Met 279	TM6	6.41	0.265	0.265	0.261	0.264	0.264	0.264	0.243
Thr 281	TM6	6.43	0.259	0.265	0.264	0.257	0.267	0.269	0.249
Phe 282	TM6	6.44	0.297	0.297	0.292	0.296	0.295	0.292	0.283
Thr 283	TM6	6.45	0.257	0.259	0.253	0.270	0.259	0.263	0.265
Leu 284	TM6	6.46	0.243	0.244	0.257	0.243	0.246	0.250	0.240
Cys 285	TM6	6.47	0.267	0.267	0.271	0.266	0.265	0.270	0.265
Trp 286	TM6	6.48	0.290	0.285	0.294	0.284	0.291	0.288	0.297
Leu 287	TM6	6.49	0.250	0.248	0.251	0.246	0.251	0.257	0.253
Phe 289	TM6	6.51	0.249	0.250	0.262	0.248	0.254	0.261	0.259
Phe 290	TM6	6.52	0.257	0.255	0.261	0.252	0.260	0.261	0.270
Asn 312	TM7	7.39	0.260	0.263	0.260	0.264	0.263	0.264	0.268
Ile 314	TM7	7.41	0.247	0.253	0.253	0.251	0.256	0.258	0.260
Gly 315	TM7	7.42	0.255	0.266	0.261	0.266	0.269	0.265	0.267
Tyr 316	TM7	7.43	0.281	0.290	0.287	0.285	0.292	0.284	0.287
Val 317	TM7	7.44	0.255	0.260	0.259	0.262	0.264	0.258	0.268
Asn 318	TM7	7.45	0.269	0.288	0.280	0.275	0.291	0.281	0.284
Ser 319	TM7	7.46	0.282	0.280	0.278	0.272	0.279	0.277	0.285
Gly 320	TM7	7.47	0.256	0.254	0.254	0.253	0.255	0.256	0.241
Phe 321	TM7	7.48	0.259	0.252	0.255	0.254	0.253	0.262	0.260
Asn 322	TM7	7.49	0.282	0.269	0.267	0.268	0.277	0.280	0.278
Pro 323	TM7	7.50	0.266	0.259	0.263	0.254	0.260	0.257	0.252
Tyr 326	TM7	7.53	0.264	0.260	0.263	0.264	0.259	0.264	0.268

a) Color code – green: above cutoff value; gray: below the cutoff value.

Supplementary Table 2. Residues for which the difference in centrality exceeded cutoff values set to the average plus or minus 2.5 times the standard deviation across all residues (cutoff values 0.022 and –0.026).

			Difference in Centrality [a]					
Residue	Domain	GPCR ident.	3POG - 2RH1	3POG - 3D4S	3POG - 3NY8	3POG - 3NY9	3POG - 3NYA	3POG - 3PDS
Trp 32	TM1	1.31	0.002	0.002	0.016	0.024	0.024	0.015
Pro 138	IL2		0.027	0.028	0.023	0.030	0.015	0.028
Ser 143	IL2		-0.026	-0.023	-0.022	-0.022	-0.023	-0.025
Leu 266	IL3		-0.025	-0.032	-0.030	-0.030	-0.032	-0.024
Lys 267	TM6	6.29	-0.027	-0.031	-0.030	-0.027	-0.030	-0.026
Glu 268	TM6	6.30	-0.027	-0.029	-0.028	-0.026	-0.028	-0.027
His 269	TM6	6.31	-0.025	-0.029	-0.030	-0.024	-0.028	-0.032
Lys 270	TM6	6.32	-0.030	-0.034	-0.029	-0.029	-0.033	-0.032
Ala 271	TM6	6.33	-0.028	-0.034	-0.032	-0.028	-0.032	-0.034
Leu 272	TM6	6.34	-0.033	-0.038	-0.036	-0.033	-0.036	-0.035
Lys 273	TM6	6.35	-0.024	-0.027	-0.027	-0.023	-0.026	-0.031
Thr 274	TM6	6.36	-0.034	-0.037	-0.033	-0.035	-0.036	-0.035
Leu 275	TM6	6.37	-0.036	-0.043	-0.041	-0.036	-0.038	-0.037

a) Color code – green: above the positive cutoff value; red: below the negative cutoff value; gray: not beyond the cutoff values.

Supplementary Table 3. Residues for which the difference in centrality was higher than a cutoff value set to the average plus 2.5 times the standard deviation across all residues (cutoff value 0.015).

			Difference in Centrality [a]						
**Residue**	**Domain**	**GPCR ident.**	**2RH1**	**3D4S**	**3NY8**	**3NY9**	**3NYA**	**3PDS**	**3P0G**
Val 33	TM1	1.32	0.002	0.002	0.002	0.002	0.001	0.022	0.003
Gly 90	TM2	2.61	0.002	0.003	0.005	0.004	0.001	0.021	0.001
His 93	TM2	2.64	0.007	0.004	0.004	0.005	0.004	0.024[b]	0.022
Ile 94	TM2	2.65	0.001	0.001	0.009	0.001	0.007	0.032	0.005
Leu 95	TM2	2.66	0.000	0.000	0.004	0.000	0.004	0.017	0.004
Met 96	TM2	2.67	0.001	0.001	0.002	0.001	0.001	0.019	0.013
Lys 97	EL1		0.003	0.003	0.003	0.003	0.002	0.043	0.011
Met 98	EL1		0.004	0.003	0.003	0.003	0.003	0.015	0.015
Trp 99	EL1		0.006	0.005	0.004	0.006	0.004	0.029	0.003
Thr 100	EL1		0.005	0.004	0.003	0.004	0.003	0.017	0.002
Val 117	TM3	3.36	0.004	0.005	0.006	0.006	0.008	0.017	0.007
Thr 118	TM3	3.37	0.014	0.016	0.016	0.016	0.017	0.008	0.001
Glu 122	TM3	3.41	0.007	0.010	0.011	0.010	0.012	0.018	0.012
Ala 176	EL2		0.014	0.019	0.000	0.020	0.018	0.012	0.000
His 178	EL2		0.015	0.019	0.001	0.019	0.018	0.015	0.000
Glu 180	EL2		0.000	0.001	0.000	0.001	0.000	0.000	0.018
Tyr 185	EL2		0.000	0.001	0.000	0.000	0.000	0.009	0.016
Glu 188	EL2		0.004	0.003	0.003	0.004	0.002	0.017	0.003
Cys 191	EL2		0.000	0.019	0.016	0.002	0.015	0.017	0.015
Asp 192	EL2		0.002	0.002	0.000	0.001	0.000	0.020	0.026
Thr 195	EL2		0.022	0.028	0.002	0.028	0.028	0.024	-0.001
Tyr 199	TM5	5.38	0.012	0.011	0.013	0.012	0.013	0.020	0.015
Ala 200	TM5	5.39	0.020	0.021	0.023	0.021	0.020	0.029	0.014
Ser 203	TM5	5.42	0.018	0.018	0.020	0.018	0.020	0.016	0.012
Ser 204	TM5	5.43	0.011	0.016	0.016	0.014	0.014	0.022	0.019
Ile 205	TM5	5.44	0.009	0.010	0.012	0.009	0.010	0.015	0.013
Ser 207	TM5	5.46	0.013	0.010	0.011	0.012	0.015	0.025	0.015
Phe 208	TM5	5.47	0.000	0.016	0.002	0.000	0.002	0.008	0.005
Pro 211	TM5	5.50	0.006	0.008	0.007	0.007	0.009	0.017	0.013

Trp 286	TM6	6.48	0.007	0.009	0.005	0.009	0.009	0.013	0.015
Phe 290	TM6	6.52	0.007	0.005	0.005	0.006	0.005	0.016	0.015
Asn 293	TM6	6.55	0.010	0.015	0.007	0.014	0.003	0.015	0.003
His 296	TM6	6.58	0.000	0.002	0.002	0.032	0.001	0.001	-0.001
Val 297	TM6	6.59	0.006	0.009	0.005	0.037	0.003	0.012	0.004
Gln 299	EL3		0.000	0.001	0.001	0.019	0.001	0.000	0.000
Asp 300	EL3		0.000	0.002	0.001	0.017	0.001	0.001	0.000
Lys 305	TM7	7.32	0.006	0.006	0.005	0.006	0.006	0.036	0.018
Ile 309	TM7	7.36	-0.001	0.000	0.016	0.000	0.015	0.012	0.010
Trp 313	TM7	7.40	0.005	0.005	0.005	0.005	0.004	0.021	0.019

a) Color code – green: above the positive cut-off value; red: below the negative cutoff value; gray: not beyond the cutoff values; residues beyond the cutoff values are highlighted in yellow for the blocker-bound structures and in cyan for the agonist-bound structures.

b) The FAUC 50 ligand is covalently bound to a Cys residue artificially introduced in place of His 93$^{2.64}$

Supplementary Figure 1. Panel a: visual representation of the interaction between one of the carboxylate oxygen atoms of Asp $113^{3.32}$ and the hydroxyl group of Tyr $316^{7.43}$, detected in the 2RH1 structure – the side chains of the two residues are shown in sticks format, with the carbon atoms colored in gray, oxygen atoms in red, and hydrogen atoms in white. Panel b: the CSU output for the interaction shown in panel a. Panel c: the same interaction represented in the form of a Python dictionary. Panel d: Python dictionary describing all the atomic interactions that connect Asp $113^{3.32}$ and Tyr $316^{7.43}$. Each atomic interaction is encoded in the form of a separate python dictionary, like the one shown in panel c.

b. CSU output line describing one interaction between Asp 113 and Tyr 316.

```
ASP  113   A CG    7.6 136.8 TYR  316   A OH   3.7  1.1 6 1
```

c. Corresponding Python dictionary describing the same interaction.

```
 {
   "Atom1Name": "OH",
   "Residue1Name": "TYR",
   "Residue1Number": 316,
   "Residue1Chain": "A",
   "AccessibleSurface": 5.0,
   "Distance": 2.7,
   "ContactSurface": 18.2,
   "FreeAccessibleSurface": 105.7,
   "Residue2Number": 113,
   "Atom2Name": "OD2",
   "Atom1Type": 1,
   "Atom2Type": 2,
   "Residue2Name": "ASP",
   "Residue2Chain": "A"
 }
```

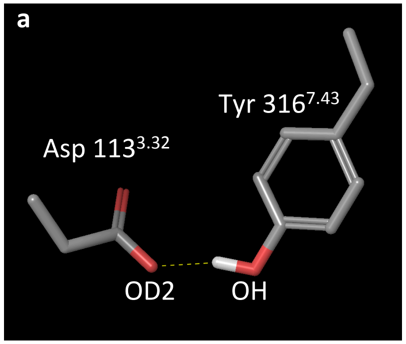

d. Python dictionary listing all the interactions between Asp 113 and Tyr 316.

```
“Tyr316”: {
    “Asp113”: [
        Interaction between Tyr316 CE2 and Asp113 OD2,
        Interaction between Tyr316 CE2 and Asp113 OD1,
        Interaction between Tyr316 CZ and Asp113 OD2,
        Interaction between Tyr316 OH and Asp113 OD2,
    ]
}
```

Supplementary Figure 2. Amino acid sequence of the seven β_2 AR structures subjected to the analysis. The figure shows only the residues solved in all seven structures, since those solved only in some of the structures were not included in our analysis. Specifically, the sequence segments included in the analysis are two: the first one (residues 32- 227) spans the entire region from the beginning of TM1 to the end of TM5; the second one (residues 266-342) spans the entire region from the beginning of TM6 to the end of the C-terminal helix 8. The N-terminus (residues 1-31), the third intracellular loop (residues 228-265), and the C-terminus beyond helix 8 residues (343- 413) were not included in the analysis. The domains are indicated with bold labels placed under the sequence and the seven TMs are highlighted in gray.

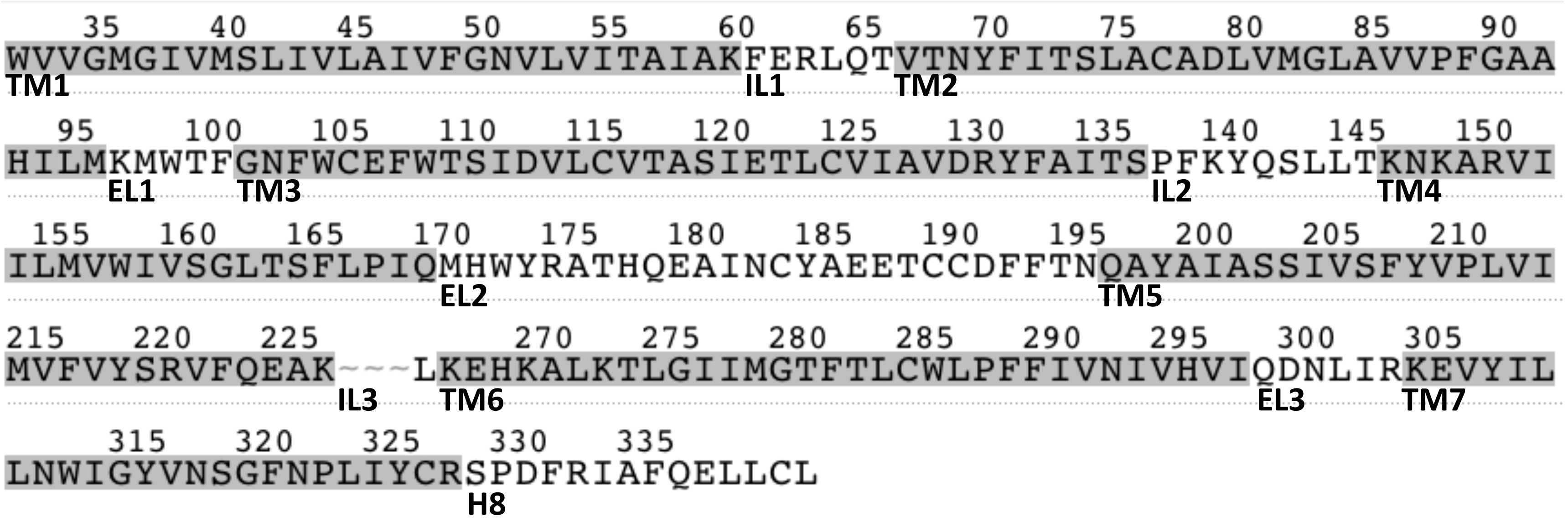

Supplementary Figure 3. Three-dimensional representation of the β2 AR, showing the residues for which the difference in closeness-centrality with respect of the activated structure (3P0G) exceeds the average plus 2.5 times the standard deviation of the values detected across all residues, for the 2RH1 (panel a), 34DS (panel b), 3NY8 (panel c), 3NY9 (panel d), 3NYA (panel e), and 3PDS (pane f) structures. For all the structures, the region in which most of the differences are concentrated is located toward the cytosolic end of TM6. For details on the graphical representation of the structures, see the legend of **Figures 5**.

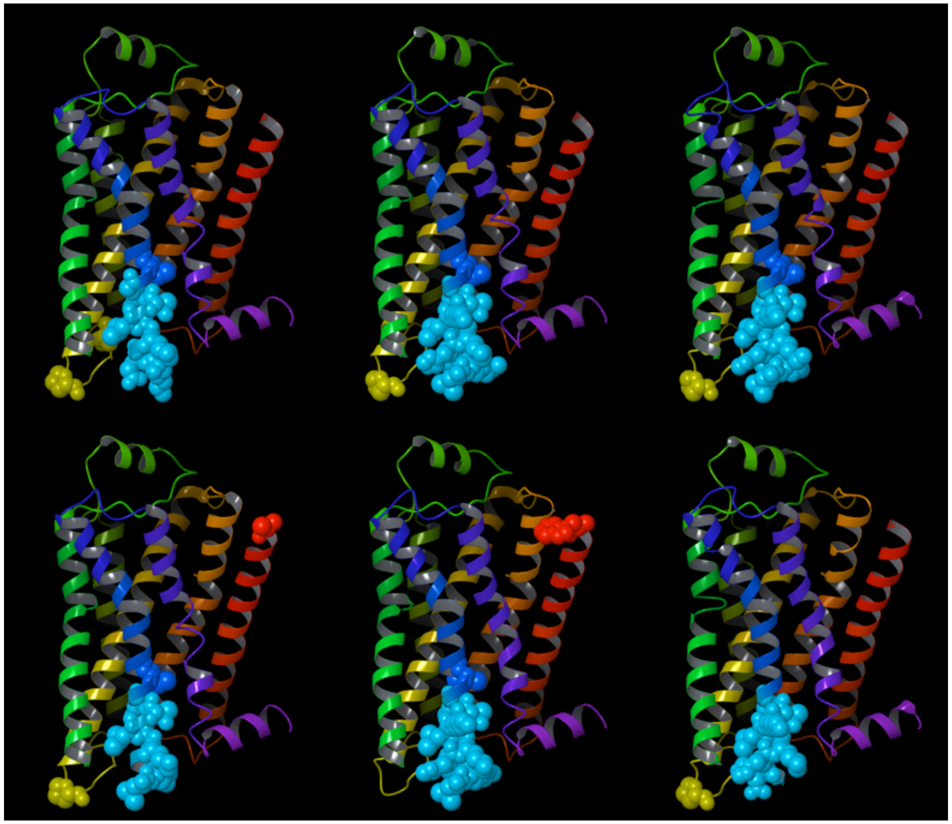

Supplementary Figure 4. Three-dimensional representation of the β2 AR, showing the residues for which the difference in closeness-centrality detected when the analyses were performed in the presence *versus* the absence of ligands exceeded the average plus 2.5 times the standard deviation of the values detected across all residues, for the blocker-bound structures 2RH1 (panel a), 34DS (panel b), 3NY8 (panel c), 3NY9 (panel d), 3NYA (panel e). Equivalent figures for the agonist-bound structures 3PDS and 3POG are given in panels c and d of **Figure 9**. For details on the graphical representation of the structures, see the legend of **Figure 5**.

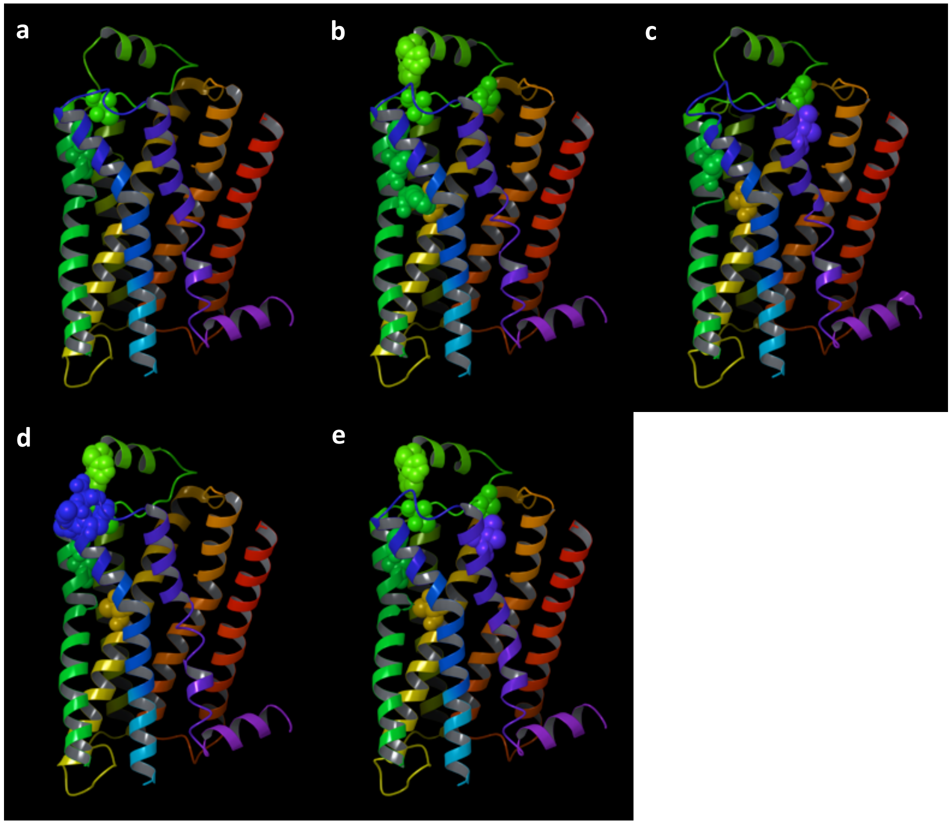

Supplement: Supplementary file 1 — Additional file 1: Table S1: Residues showing the highest network centrality; Table S2. Difference in network closeness-centrality with respect to 3POG; Table S3. Change in network centrality when the structures are analyzed with or without ligands; Figure S1. Example showing how non-covalent interactions are stored in Python dictionary. Figure S2. Amino acid sequence of the β2 AR structures subjected to the analysis; Figure S3. The six individual structures that are shown superimposed in Figure 10; Figure S4. The five individual structures that are shown superimposed in panel a of Figure 12. (PDF 1 MB) [file 40203_2013_18_MOESM1_ESM.pdf]
